# Depletion of mucin in mucin-producing human gastrointestinal carcinoma: Results from *in vitro* and *in vivo* studies with bromelain and N-acetylcysteine

**DOI:** 10.18632/oncotarget.5259

**Published:** 2015-09-30

**Authors:** Afshin Amini, Samar Masoumi-Moghaddam, Anahid Ehteda, Winston Liauw, David L. Morris

**Affiliations:** ^1^ Department of Surgery, St George Hospital, The University of New South Wales, Kogarah, Sydney NSW 2217, Australia; ^2^ Cancer Care Center, St George Hospital, The University of New South Wales, Kogarah, Sydney NSW 2217, Australia

**Keywords:** bromelain, gastrointestinal cancers, mucin, mucin-depleting effect, N-acetylcysteine

## Abstract

Aberrant expression of membrane-associated and secreted mucins, as evident in epithelial tumors, is known to facilitate tumor growth, progression and metastasis, and to provide protection against adverse growth conditions, chemotherapy and immune surveillance. Emerging evidence provides support for the oncogenic role of MUC1 in gastrointestinal carcinomas and relates its expression to an invasive phenotype. Similarly, mucinous differentiation of gastrointestinal tumors, in particular increased or *de novo* expression of MUC2 and/or MUC5AC, is widely believed to imply an adverse clinicopathological feature. Through formation of viscous gels, too, MUC2 and MUC5AC significantly contribute to the biology and pathogenesis of mucin-secreting gastrointestinal tumors. Here, we investigated the mucin-depleting effects of bromelain (BR) and N-acetylcysteine (NAC), in nine different regimens as single or combination therapy, in *in vitro* (MKN45, KATOIII and LS174T cell lines) and *in vivo* (female nude mice bearing intraperitoneal MKN45 and LS174T) settings. The inhibitory effects of the treatment on cancer cell growth and proliferation were also evaluated *in vivo*. Our results suggest that a combination of BR and NAC with dual effects on growth and mucin products of mucin-expressing tumor cells is a promising candidate towards the development of novel approaches to gastrointestinal malignancies with the involvement of mucin pathology. This capability supports the use of this combination formulation in locoregional approaches for reducing the adverse effects of the aberrantly secreted gel-forming mucins, as in pseudomyxoma peritonei and similar pathologies with ectopic production of mucin.

## INTRODUCTION

Mucins are a diverse family of high molecular weight, heavily glycosylated proteins that are differentially expressed by specialized epithelial cells of mucosal and secretory surfaces throughout the body in a relatively organ- and cell type-specific manner [1]. The structural characteristics of mucin are primary indicators of its physiological function and changes to its composition have been identified in gastrointestinal pathologies, including gastric and colorectal cancer [2]. Moreover, mucins have long been implicated in the pathogenesis of cancer. As sites of tumor growth are often hypoxic, acidic and laden with proteases and other biologically active factors, it is possible that tumors use mucins to configure the local microenvironment during invasion, metastasis and growth in sites and conditions that might be inhospitable [3]. Furthermore, mucins evidently make individual contributions to the biology and clinical features of some peritoneal malignancies, including peritoneal carcinomatosis (PC) from mucinous gastrointestinal adenocarcinomas and pseudomyxoma peritonei (PMP) [4]. In the past three decades cytoreductive surgery (CRS) and hyperthermic intraperitoneal chemotherapy (HIPEC) has emerged as a useful treatment for the management of this challenging entity [5, 6]. In order to maintain a disease-free peritoneal surface after complete cytoreduction, additional efforts should be made to optimize, and even customize, HIPEC and to improve locoregional treatment. For this purpose, the efficacy of HIPEC needs to be enhanced not only by determining optimal agents, and duration and temperature of hyperthermia, but also by developing novel locoregional treatments.

Bromelain (BR) and N-acetylcysteine (NAC) are two natural agents with good safety profiles shown to have anti-cancer effects. Previously, we reported the capability of BR/NAC, in particular in combination, in significantly inhibiting the growth and proliferation of a panel of gastrointestinal cancer cells [7]. Taking into consideration the significance of the expression of mucins as contributory factors in the pathophysiology of cancer, we intended in this study to explore another feature of the treatment, that is to say whether BR/NAC treatment could alter the expression of mucin by gastrointestinal cancer cells employing *in vitro* and *in vivo* models.

## RESULTS

### BR/NAC treatment decreased PAS-stained mucosubstances

**Figure 1:**
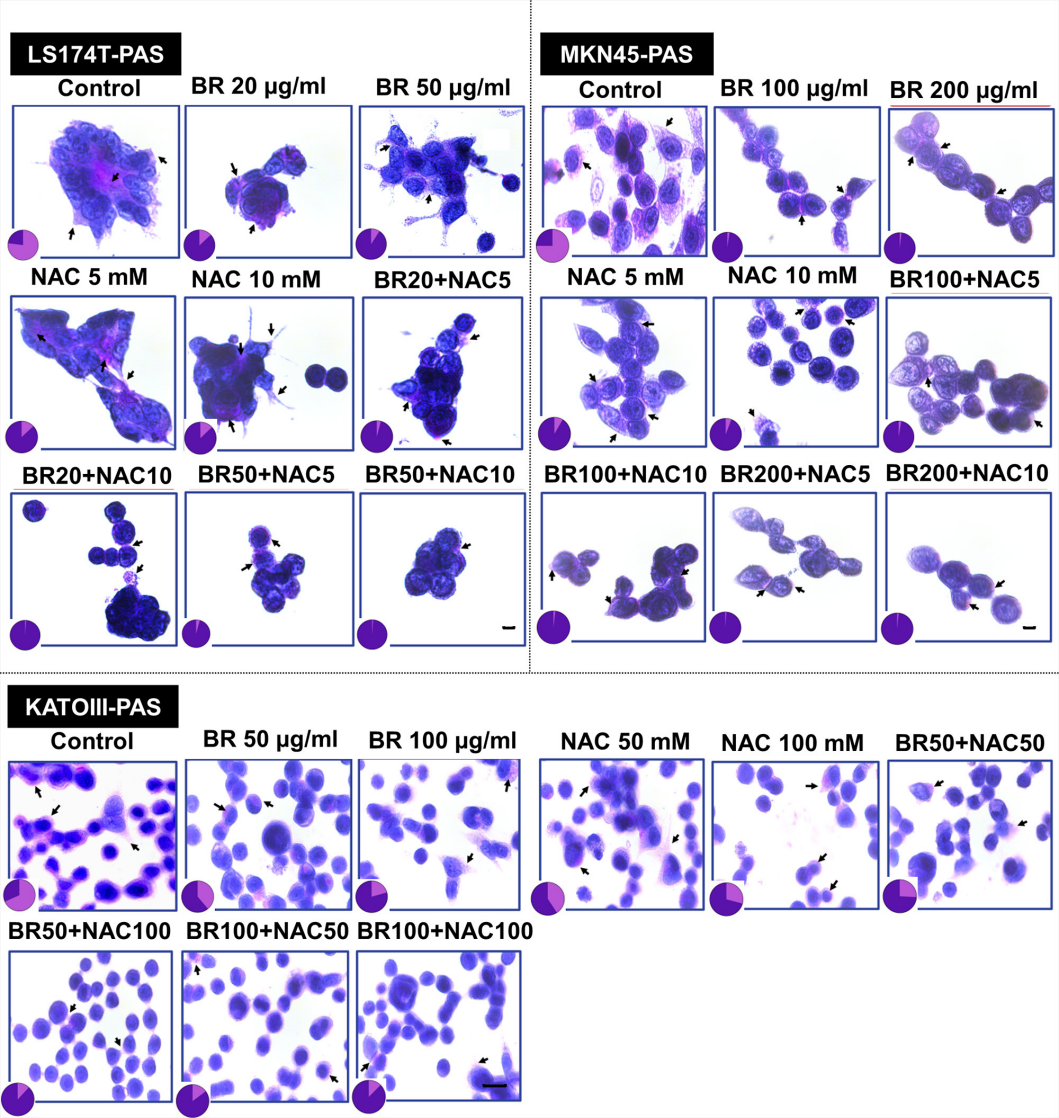
Periodic Acid-Schiff's (PAS) staining of gastrointestinal cancer cells Representative photos demonstrate PAS positive mucosubstances stained rose to magenta with blue nuclei. The PAS-stained areas decreased after 48 hours of treatment with bromelain, NAC or the combination. Pie graphs compare PAS-positive areas (pink segment) vs PAS-negative areas (purple segment). Scale bar: 50 μm.

The effect of BR/NAC treatment on the production of mucosubstances was assessed using PAS staining. As a result of treatment, the amount of the PAS-positive substances was dramatically reduced in response to BR and NAC in MKN45 cells. The decrease in PAS-stained area was more prominent in BR and combination treatment groups; in particular in the latter where the amount of the remaining mucosubstances was minimal. As expected, similar results were obtained when LS174T and KATOIII cells were employed. Figure 1 demonstrates and compares the staining status in treated groups as compared with untreated control, indicating that BR/NAC treatment remarkably reduced the PAS-stained areas and that mucosubstances were barely detected in combination groups.

### Reduced immunocytochemical expression of MUC1, MUC2 and MUC5AC was evident following BR/NAC treatment

**Figure 2:**
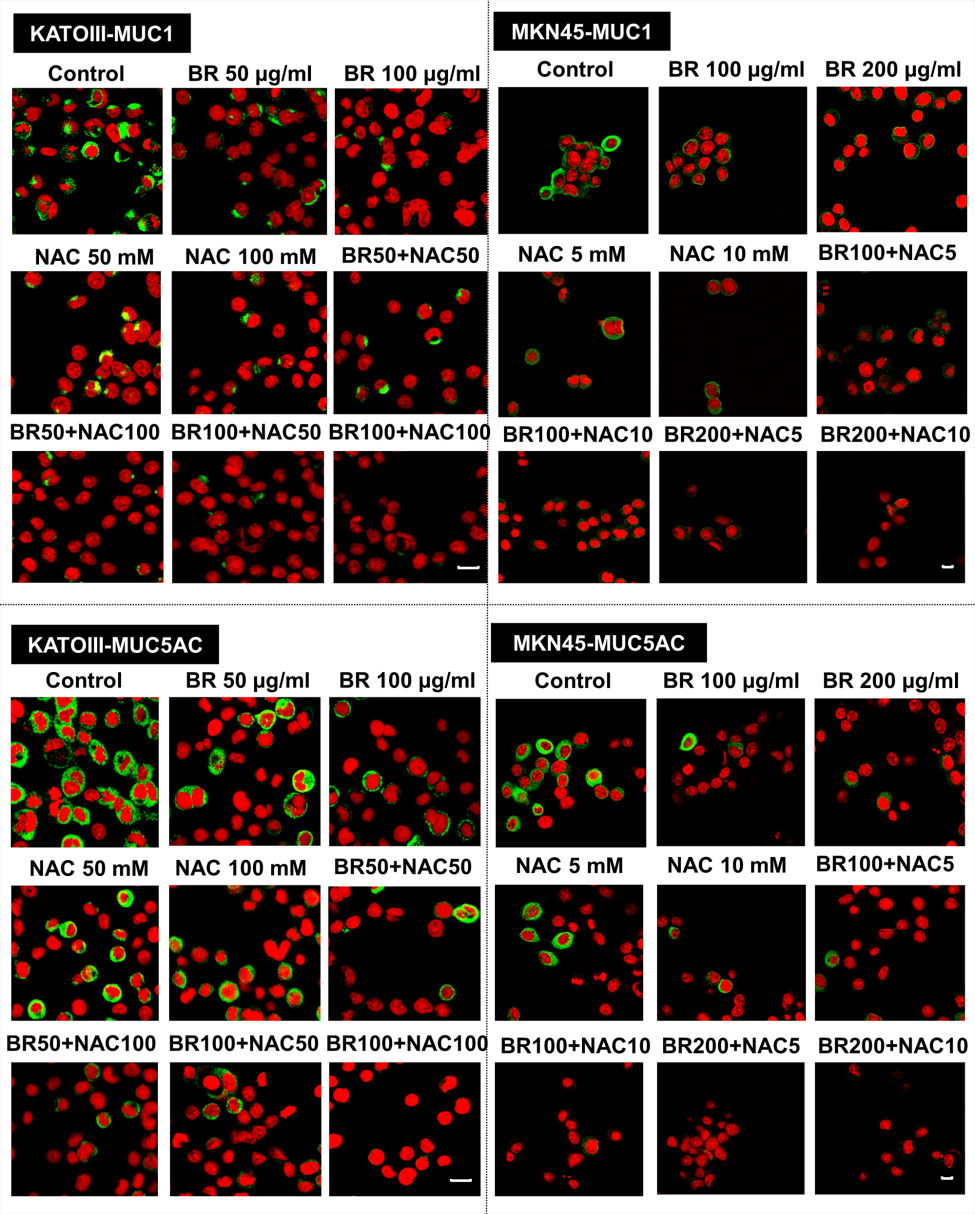
Immunofluorescence staining of gastric cancer cells After 48 hours of treatment with bromelain, NAC or the combination, cells were assayed for MUC1 and MUC5AC expression and viewed under laser scanning confocal microscope. Green and red fluorescence correspond to MUC and the nucleus, respectively, indicating the reduction in mucin expression of gastric cells in all treatment groups as compared with untreated control cells. Scale bar: 50 μm.

**Figure 3:**
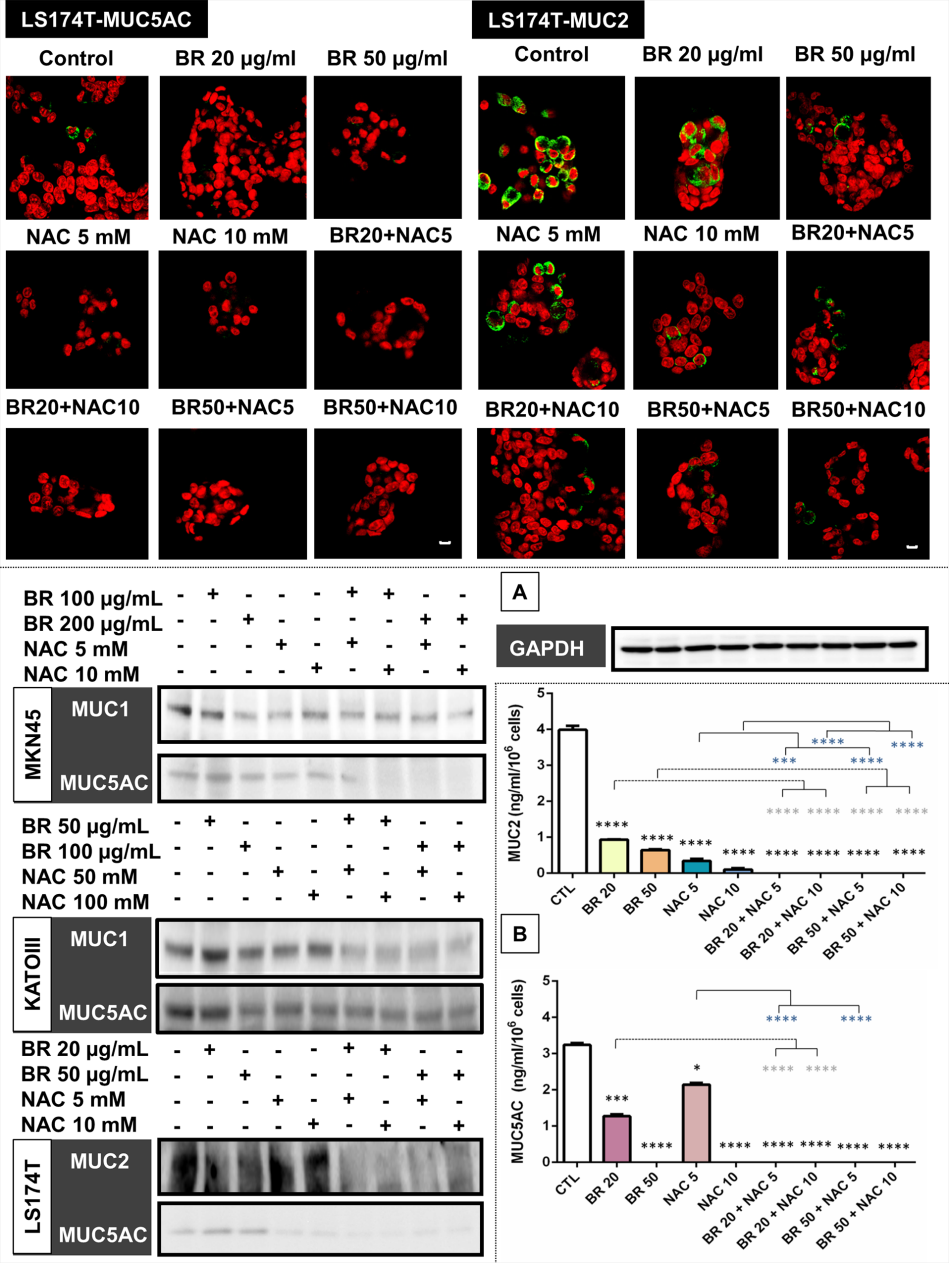
Immunofluorescence staining of colon cancer cells (upper micrographs), western blot analysis of the expression of mucin proteins in gastrointestinal cancer cells (A) and ELISA assay of secreted mucins levels in media bathing LS174T colon cells (B) after 48 hours of treatment with bromelain, NAC or the combination LS174T cells were assayed for MUC2 and MUC5AC expression and analyzed under laser scanning confocal microscope. Green and red fluorescence correspond to MUC and the nucleus, respectively, indicating the reduction in mucin expression of the cells in all treatment groups as compared with untreated control cells. Scale bar: 50 μm. **A.** Representative photos demonstrate decreased expression of MUC1, MUC2 and MUC5AC; **B.** The graphs show a reduction in the amount of secretory mucin in LS174T cells media after treatment. The values shown are mean ± SE of data from three independent experiments (*p < 0.05, ***p < 0.001 and ****p < 0.0001 vs. control group). Asterisks in gray and blue colors represent the results of analysis when either dosages of single bromelain or NAC was considered as control vs their relevant combination. Bromelain, μg/mL; NAC, mM.

BR/NAC treated cells and their untreated controls were subjected to immunocytochemical analysis to specify the results for different types of mucin probed by their specific antibodies. Employing MKN45 and KATOIII, we observed strong expression of MUC1 in control group with cytoplasmic localization. However, it was found to be reduced after BR/NAC treatment (Figure 2, top). This feature was more prominent in association with combination therapy. As anticipated, LS174T cells did not appear to express MUC1 in a similar experiment. With regard to MUC5AC, untreated MKN45, KATOIII (Figure 2, bottom) and LS174T cells (Figure 3, top) strongly expressed cytoplasmic MUC5AC. In contrast, decreased expression of MUC5AC was evident in treated cells. Consistent with the earlier observations, an even more remarkable decrease in MUC5AC expression resulted from combined use of BR and NAC. The immunocytochemical expression of MUC2 in LS174T cells demonstrated the strong cytoplasmic pattern which was diminished by BR/NAC treatment (Figure 3, top). The reduction in MUC2 expression was more prominent in combination therapy.

### BR/NAC attenuated the MUC1, MUC2 and MUC5AC contents of the cells

Following immunocytochemical study on MUC glycoproteins expressed in the three models, we intended to confirm the results by Western blot analysis. As seen, BR/NAC treatment reduced the expression of MUC1 and MUC5AC in MKN45 and KATOIII cells as well as of MUC2 and MUC5AC in LS174T (Figure 3-A). On average, combination therapy more significantly affected mucin expression than did single agent treatment.

### BR/NAC reduced LS174T secretion of MUC2 and MUC5AC into culture media

Culture media levels of the secreted mucins produced by treated and untreated LS174T cells were examined and compared by ELISA. As seen in Figure 3-B, BR/NAC treatment significantly decreased culture media levels of the both secreted mucins. This effect was more prominent with BR/NAC combination treatment, with MUC2 and MUC5AC being undetectable in all combination groups.

### BR/NAC reduced tumor burden in two nude mouse models of PC

**Figure 4:**
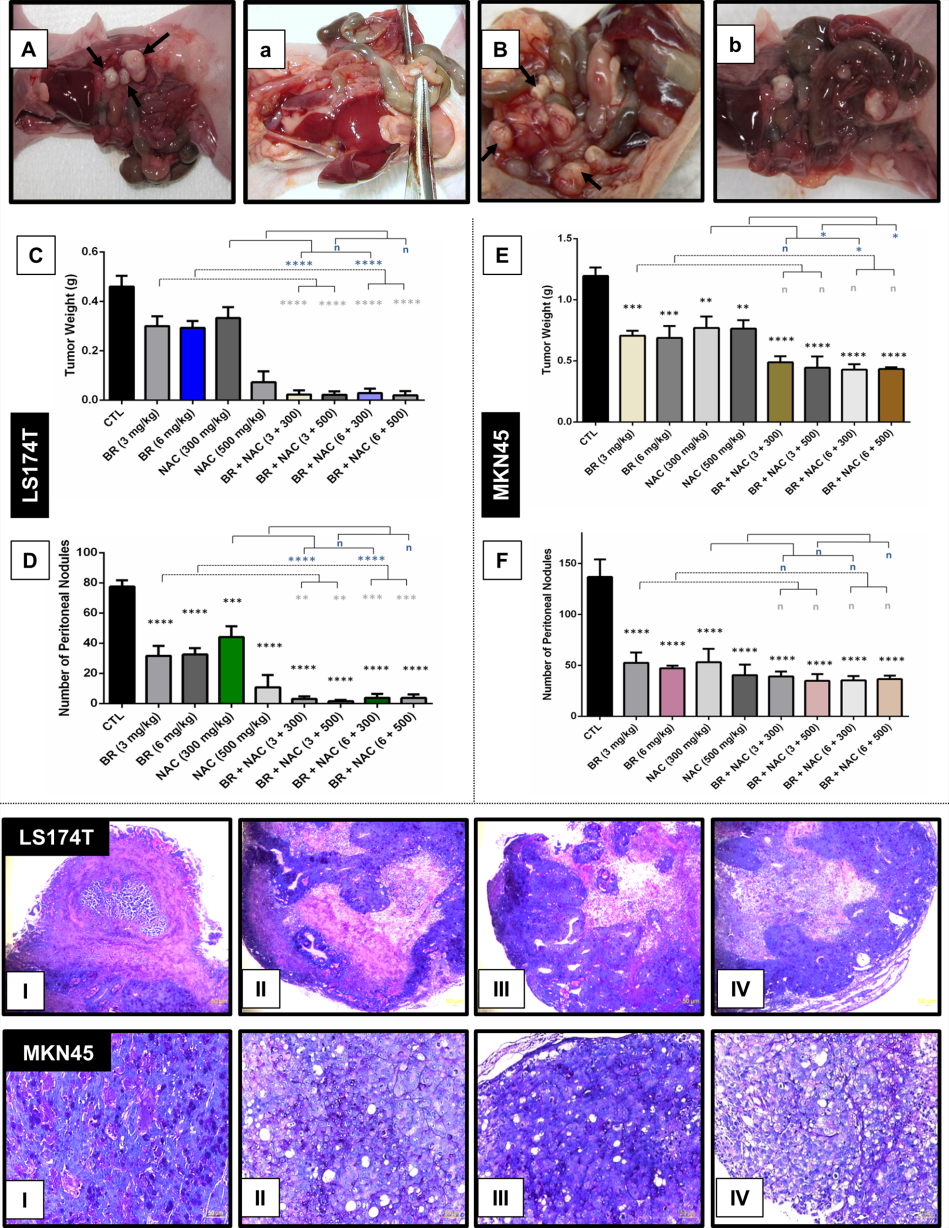
Inhibitory effects of intraperitoneal administration of BR and NAC on tumor burden and mucin products of the *in vivo* models of gastrointestinal cancer **A–B.** represent untreated controls of LS174T and MKN45 models, respectively; (a–b), represent high-dose BR and NAC combination treatment group in LS174T and MKN45 xenografts, respectively; **C–E.** graphs show tumor weight and **D–F.** demonstrate the number of peritoneal nodules decreased in a dose dependent fashion. The values shown are mean ± SE of data (**P < 0.01, ***p < 0.001 and ****p < 0.0001, n: non-significant vs. control group). Asterisks in gray and blue colors represent the results of analysis when either dosages of single bromelain or NAC was considered as control vs their relevant combination. Bromelain, μg/mL; NAC, mM. (I–IV), micrographs are representatives of Periodic Acid-Schiff's (PAS) staining of *in vivo* tumor sections. PAS positive mucosubstances stained rose to magenta with blue nuclei showing decreased PAS-stained contents of the samples in 6mg/kg bromelain(II), 500mg/kg NAC (III) and the combination of 6mg/kg bromelain and 500mg/kg NAC (IV) groups as compared to the untreated control (I) Scale bar: 50 μm.

Following development of two nude mice models of PC (Figure 4-A, 4-B), the efficacy of locoregional treatment with BR/NAC was evaluated *in vivo*. Our results showed that intraperitoneal administration of BR/NAC as single agent or combination treatment on alternate days inhibited tumor growth (Figure 4-C, 4-E) and the number of peritoneal nodules (Figure 4-D, 4-F) in a dose dependent fashion.

### BR/NAC decreased tumor mucosubstances in *in vivo* models

As shown in Figure 4, bottom, treatment with BR/NAC remarkably reduced the PAS-positive substances of tumor samples. The reduction in mucosubstances contents of mucin pools was more prominent in LS174T tumor model (Figure 7-A, 7-B). Moreover, areas of apoptotic bodies were observed as a result of intraperitoneal treatment (Figure 7-b).

### BR/NAC reduced tumor cell proliferation index in both nude mouse models

**Figure 5:**
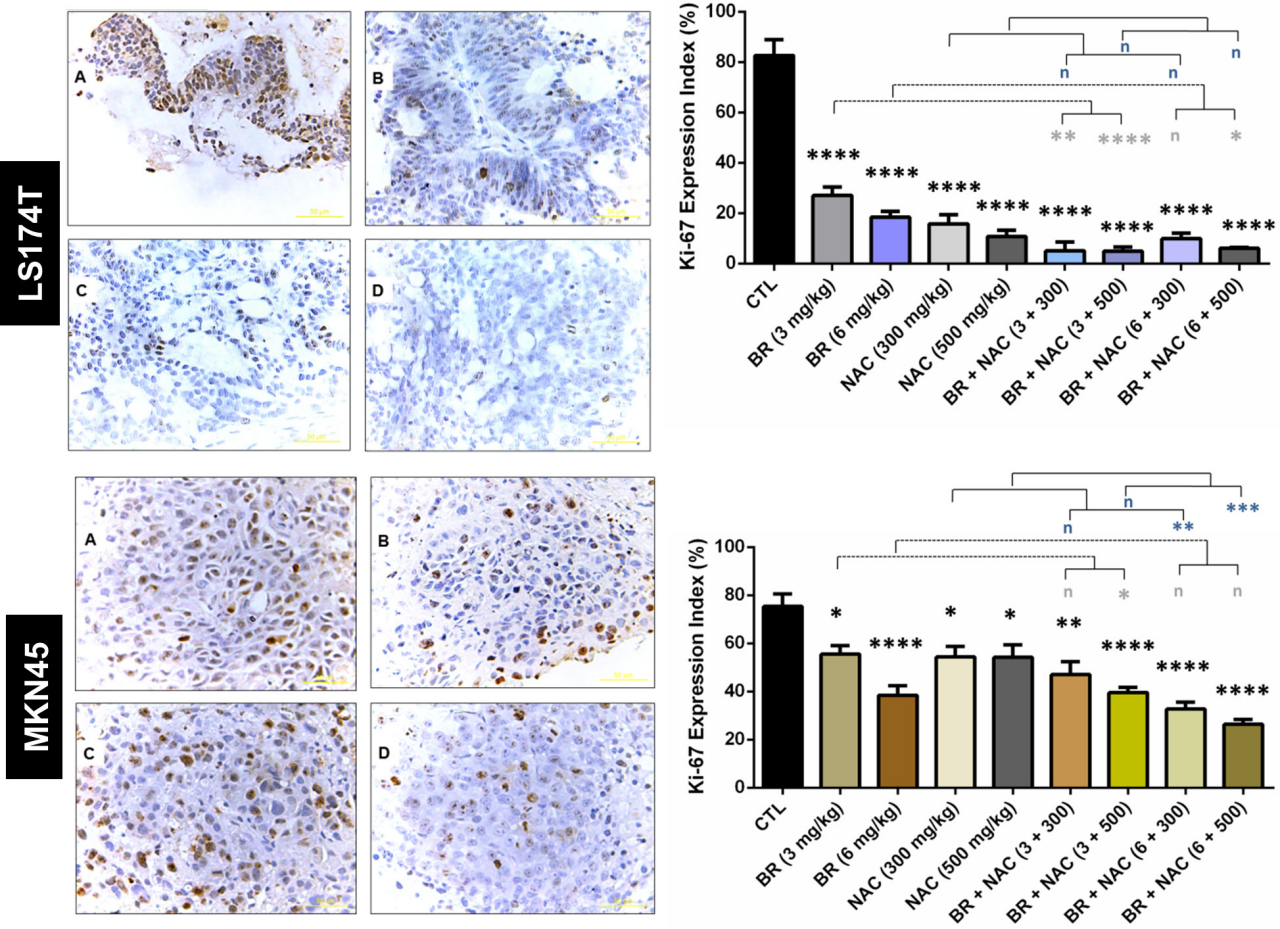
Immunohistochemical analysis of the expression of Ki-67 in the studied gastrointestinal cancer xenografts Upper and lower sections show LS174T and MKN45 xenografts, respectively, with decreased Ki-67 expression indexes as demonstrated in the right graphs. The values shown are mean ± SE of data (*p < 0.05, **P < 0.01 and ****p < 0.0001, n: non-significant vs. control group). Asterisks in gray and blue colors represent the results of analysis when either dosages of single bromelain or NAC was considered as control vs their relevant combination. A, untreated control; B, 6mg/kg bromelain; C, 500mg/kg NAC; D, 6mg/kg bromelain + 500mg/kg NAC. Scale bar: 50 μm. Bromelain, μg/mL; NAC, mM.

To find out how tumor cell proliferation is affected by BR/NAC treatment, immunohistochemistry was employed to compare the expression of Ki-67 protein in different groups of either model. As seen in Figure 5, treatment reduced Ki-67 expression, with the lowest expression observed in the combination therapy groups. This effect in LS174T model was drastic. Ki-67 index was calculated for each group as the percentage of the positively stained cells. In MKN45 model, mean Ki-67 index ranged from a minimum of 26.33% (6 mg/kg BR+500 mg/kg NAC) to a maximum of 55.57% (6 mg/kg BR) in the treatment groups, compared with 75.33% in the untreated control group. As regards LS174T model, mean Ki-67 index of the treatment groups ranged between 4.9% (300 mg/kg BR+500 mg/kg NAC) and 27% (300 mg/kg BR), indicating a dramatic reduction in the Ki-67 expression of the treatment groups compared with control (82.6%). While all treatment regimens were found to significantly decrease Ki-67 index, combination therapy appeared significantly more effective than single agent therapy.

### BR/NAC treated mice expressed less MUC1, MUC2 and MUC5AC in their tumor tissues

**Figure 6:**
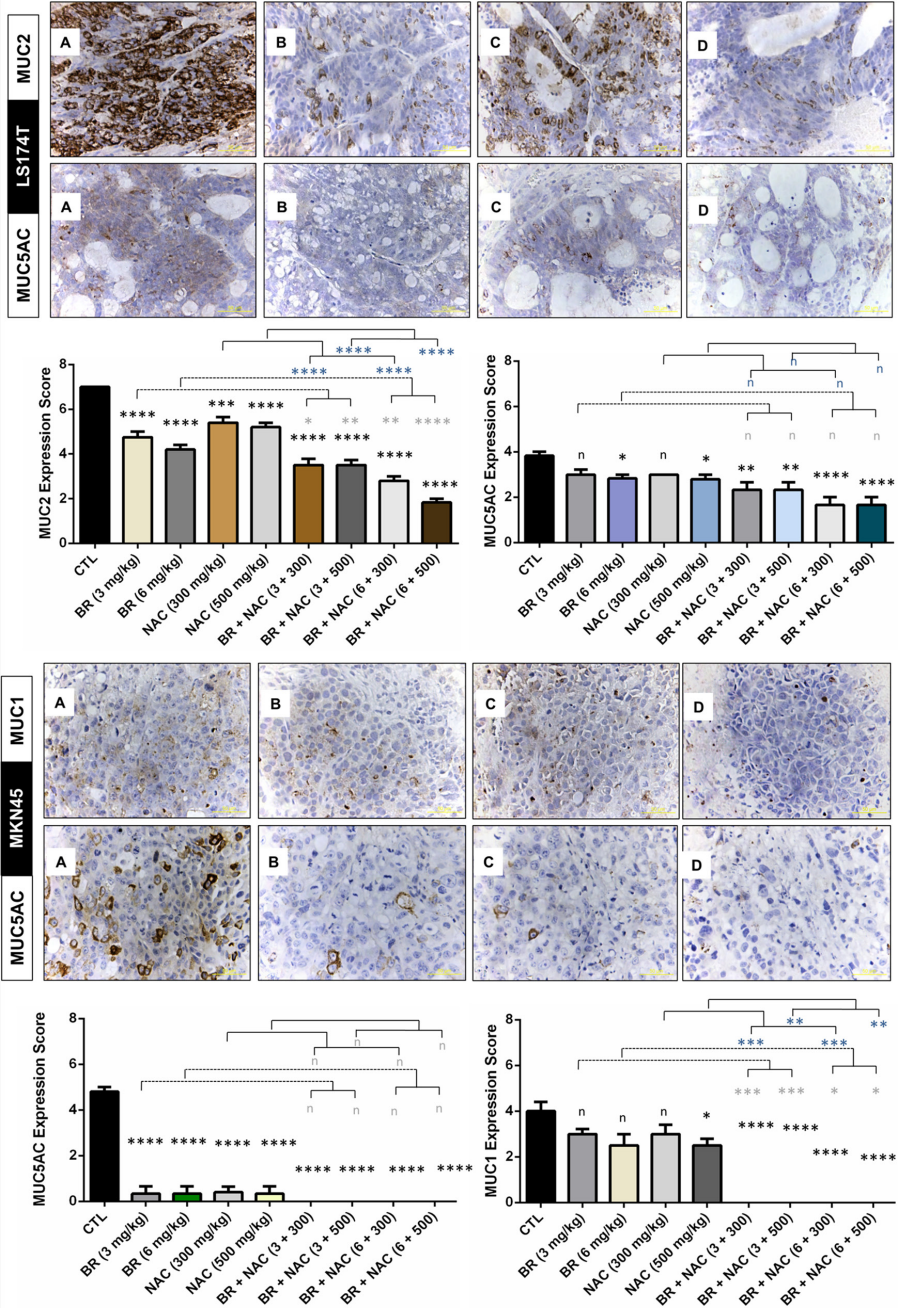
Immunohistochemical analysis of the expression of MUC1, MUC2 and MUC5AC in the studied gastrointestinal cancer xenografts Micrographs are representative photos of untreated control **A.** 6mg/kg bromelain **B.** 500mg/kg NAC **C.** and 6mg/kg bromelain + 500mg/kg NAC **D.** Scale bar: 50 μm. The graphs demonstrate immunohistochemical scores. The values shown are mean ± SE of data (*p < 0.05, **P< 0.01, ***p < 0.001 and ****p < 0.0001, n: non-significant vs. control group). Asterisks in gray and blue colors represent the results of analysis when either dosages of single bromelain or NAC was considered as control vs their relevant combination. Scale bar: 50 μm.

Performing immunohistochemical staining of tumor samples for specific types of mucin, we found reduced mucin expression in our studied *in vivo* models. As shown in Figure 6, BR and NAC, in particular in combination, significantly reduced the expression of MUC2 in treated LS174T mice compared with their control. In the same model, significantly diminished expression of MUC5AC was observed when high doses of single agent BR or NAC were administered as well as when combination therapy is used. In MKN45 model, MUC5AC was remarkably decreased in single agent groups and barely detected in combination groups. As regards MUC1, the decrease in MUC expression was more prominent when BR and NAC were applied in combination. In this model, although all single agent groups showed lower MUC1 expression, only the results of high dose NAC group was significant.

### No toxicity was observed in nude mice intraperitoneally treated with BR and NAC

**Figure 7:**
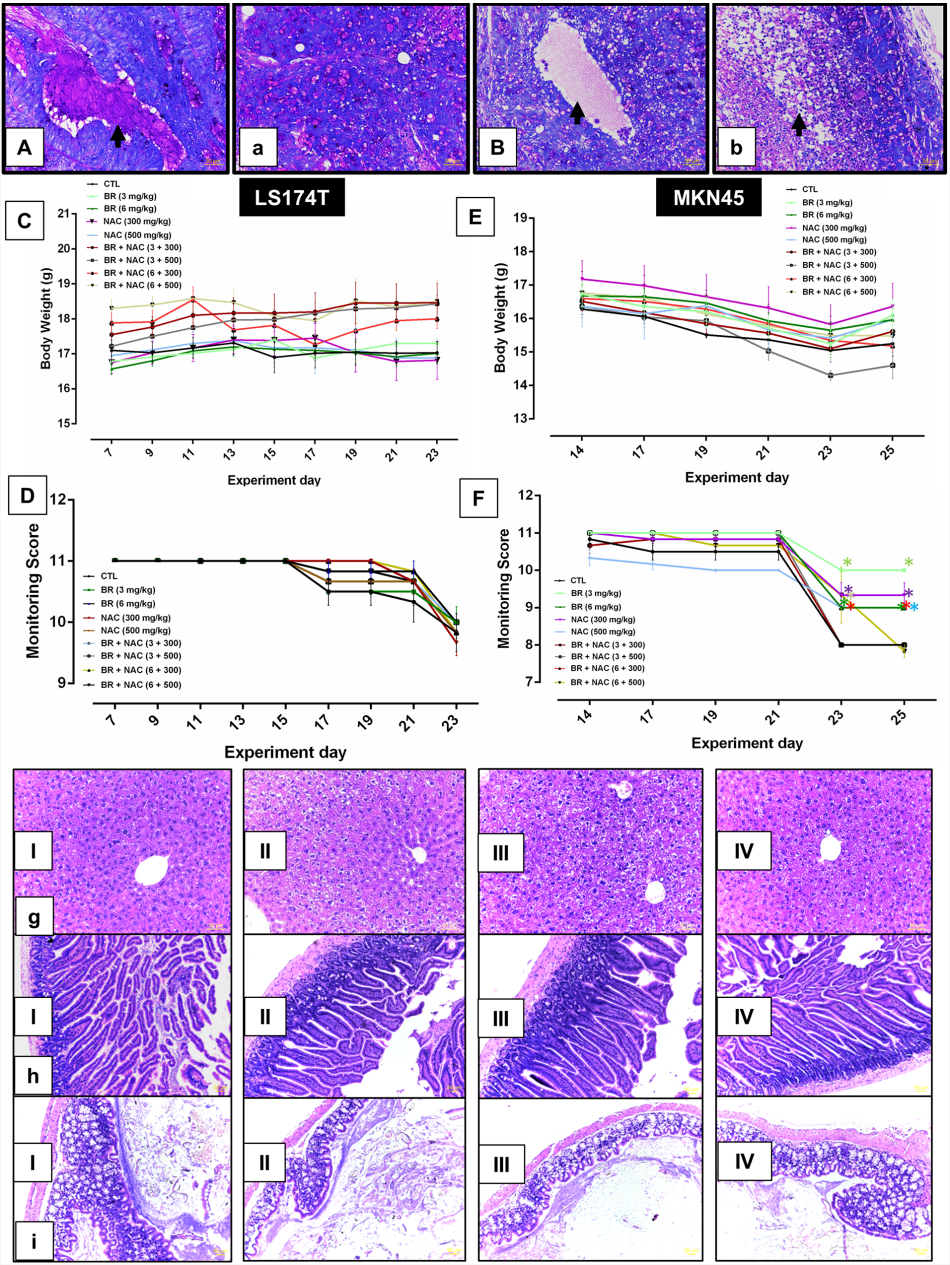
Animal toxicology studies and representative photos of Periodic Acid-Schiff's (PAS) staining of mucin pools **A.** shows a mucin pool with PAS-positive contents stained rose to magenta in LS174T tumor nodule of untreated control group as compared to **B.** which demonstrate a pool in the LS174T tumor nodule of combination group (6mg/kg bromelain + 500mg/kg NAC) with significantly decreased PAS-stained contents. a depicts small mucin pools in MKN45 tumor nodule of untreated control in comparison with b representing decreased mucin contents and apoptotic bodies (arrow) in the MKN45 tumor nodule of combination group (6mg/kg bromelain + 500mg/kg NAC). Scale bar: 50 μm. **C–F.** graphs show drug-related effects on the growth (C, E) and health monitoring scores (D, F) of rodents. The values shown are mean ± SE of data. Significant changes are shown by asterisks. I-IV micrographs represent H&E stained sections of liver g. small intestine h. and large intestine i. showing no evidence of toxic change and abnormal features in I, untreated control; II, 6mg/kg bromelain; III, 500mg/kg NAC and IV, 6mg/kg bromelain + 500mg/kg NAC. Scale bar: 50 μm.

To evaluate drug-related effects on the health and wellbeing of animals, scores obtained from health monitoring of 108 animals as well as their body weight, collected on an every-other-day basis, were employed and, were employed and pooled across relevant time points for each animal in each group. There were no treatment-related mortalities in mortalities in either group. The results showed that at the doses of BR (3, 6 mg/kg body weight) and NAC (300, 500 mg/kg body weight) examined as single or combination treatment, no significant differences in weight fluctuations were observed showing no drug-induced toxicity (Figure 7-C, 7-E). In LS174T model (Figure 7-D), BR/NAC administration did not affect health sores of the treatment groups as compared to the vehicle treated group. In MKN45 model (Figure 7-F), on days 23 and 25th of the treatment, a significant difference was observed in single BR/NAC treatment groups as well as in BR (6 mg/kg)+NAC (300 mg/kg) group compared with no-treatment group showing better health scores in the treated animals. On day 23th of treatment, BR (6 mg/kg)+NAC (500 mg/kg) also showed a significant difference in comparison with the control demonstrating higher health scores in treated mice.

At the scheduled necropsy, no treatment-related gross findings were observed in any of the treated animals. Liver appeared to have a smooth external surface with brown color at both the external and cut surfaces. In the microscopic assessment using H&E staining as the standard method in preclinical toxicology studies, no evidence of fibrosis or necrosis was present. (Figure 7-g). Treated mice also showed no gross evidence of damage in small intestine and colon tissues, such as inflammation, ulceration, wall thickening and necrosis. In H&E study of tissue sections, no microscopic damage in colon and small intestine tissue, including inflammation, ulceration, shortening of villi, crypt abscess and necrosis was observed as shown in Figure 7-h, 7-i.

## DISCUSSION

A large body of evidence indicates that the prototypical membrane-associated and secreted mucins are implicated in the pathogenesis of human carcinomas. Taking into account its apparent involvement in such key biological processes as proliferation, survival, invasion, angiogenesis, apoptosis inhibition and chemoresistance, MUC1 is believed to play critical roles in carcinogenesis and, more likely, in cancer progression and metastasis [8]. MUC2, known as the prototypical secreted mucin specifically expressed in the small intestine and colon, can be a major contributor to the pathogenesis of mucin-secreting tumors of gastric and colorectal origin and their peritoneal spread. It is the PMP-specific mucin that is responsible for the high degree of gelation. In this context, the copious secretion of MUC2 not only gives rise to the formation of the appendiceal mucocele and subsequent release of tumor cells, and allows them to freely move and redistribute within the peritoneal cavity, but also plays the key role in the development of morbidity and major complications in this condition [4]. The current data collectively supports the notion that higher expression of MUC2 and MUC5AC confers genetic and molecular features on colorectal carcinomas that incur adverse consequences in relation to treatment response and clinical outcome [9]. MUC5AC, the gastric-secreted mucin, is rarely expressed, and only by a minority of goblet cells, in the normal colon. However, it is frequently expressed in colorectal adenomas and carcinomas [10] and was shown to be strongly associated with tumorigenic features and adverse clinicopathological characteristics [11]. Both membrane-associated and secreted mucins have been implicated in the development of chemoresistance through involvement in the formation of a physical barrier, resistance to apoptosis and drug metabolism [12].

Mucolytic activity of BR and NAC, enhanced in combination therapy, holds promise for use in locoregional strategies for the management of pathological or ectopic mucin production which was previously shown in a study by our department [13]. However, this study only investigated the mucolytic effect and did not evaluate any potential effects of the treatment on mucin production and secretion. Here, we report that BR and NAC decrease production and/or secretion of mucins, in particular when the cells are exposed to combined regimens. We observed that BR/NAC significantly reduced the amount of PAS-stained substances, and showed potent mucin-depleting activity through effective reduction of the expression of MUC1, MUC2 and MUC5AC mucins *in vitro*.

NAC is a mucoactive agent with both mucolytic and mucoregulatory functions. Mucolytic activity of NAC has been evaluated in a variety of respiratory [14] and gastrointestinal tract [15–17] diseases associated with mucus hypersecretion. As a mucoregulatory agent, NAC controls mucin production and secretion in a content-dependent manner [18–24]. As discussed earlier, emerging evidence shows that MUC1 functions as an oncogene/oncoprotein and induces a chemoresistant phenotype. As the principal gastrointestinal mucins capable of forming viscous gels, MUC2 and MUC5AC can similarly play important roles in the pathogenesis and resilience of other mucin-secreting gastrointestinal tumors. Besides, they participate in the formation of an efficient barrier against chemotherapeutic drug absorption. Thus, mucin-depleted tumor cells are in fact deprived of a key biological infrastructure and a protective framework. Given the critical roles of mucins in the biology of epithelial tumors, the mucin-depleting feature of BR/NAC treatment is postulated to largely contribute to the induction of cytotoxicity [7] and chemosensitivity [25] in our *in vitro* models. Meanwhile, this capability supports the use of this formulation in locoregional approaches for reducing the adverse effects of the aberrantly secreted gel-forming mucins, as in PMP and similar pathologies with ectopic production of mucin.

For preclinical evaluation of the efficacy of BR/NAC as a candidate for locoregional treatment of gastrointestinal peritoneal surface malignancies (PSMs), two nude mice models were developed. *In vivo* models developed by MKN45 have been used as excellent models of PC from human gastric cancer with a gastric mucin phenotype [26]. In nude mice, LS174T grew as a mucinous adenocarcinoma microscopically resembling the original tumor [27] with the expression of the intestinal goblet cell-specific secreted mucins, of which MUC2 is most prominently expressed [28]. Hence, LS174T can be utilized for the development of an ideal model of PC from colorectal adenocarcinoma and a surrogate model of PMP.

In this study, intraperitoneal treatment of MKN45 tumor-bearing nude mice with BR/NAC caused significant reductions in the peritoneal tumor nodule weight and count of up to 64% and 74%, respectively. Moreover, differential effect of single agent and combination therapy on tumor weight, in favor of the latter, was statistically significant in 3 out of 8 compared pairs. These results were obtained despite the fact that the 12-day-long treatment of this animal model of gastric PC started two weeks post-inoculation, when peritoneal tumor nodules were readily visible in the primarily set up model. The depth of the drug penetration into peritoneal tumors depends on both drug and tumor type. Nevertheless, even with selected cytotoxic agents, the finite penetration depth limits the advantage of the intraperitoneal chemotherapy to patients with minimal tumor volume. Therefore, early commencement of the intraperitoneal treatment with BR/NAC, similar to intraoperative or early postoperative use of chemotherapy, is expected to offer even better results. As regards the colorectal PC model, the intraperitoneal administration of same regimens, starting one week earlier and ending 5 days later, dramatically inhibited the development of the peritoneal tumors by LS174T cells. Based on the experience with the LS174T model set-up, one-week inoculation period corresponds to the early phase of the tumor development when small peritoneal nodules form. Thus, a smaller “pre-existing tumor burden at the treatment onset” in LS174T model appears to be a major contributor to the superior efficacy of the treatment in this model in comparison with the other. This feature mimics HIPEC/EPIC targeting of “residual disease post CRS”. Besides, a more penetrable and/or sensitive tumor type (consistent with our earlier *in vitro* findings showing the differential sensitivity of MKN45 and LS174T cells to BR/NAC treatment), and a longer treatment period are postulated to play additional roles.

In addition to antimutagenic and anticarcinogenic properties, NAC has shown inhibitory effects on growth and progression of a variety of tumors *in vivo*. In a study by Albini et al on murine melanoma, the number of lung metastases decreased sharply when malignant cells inoculated were pre-treated with NAC [29]. Besides, oral treatment of mice delayed primary tumor formation and decreased tumor weights. In a subsequent study, NAC consistently exhibited similar effects in single agent therapy and demonstrated synergism with doxorubicin in combination therapy [30]. Im et al reported that NAC diminished B16-F10 tumor growth in mice after intraperitoneal treatment [31]. In an interesting study by Delneste et al, oral administration of NAC inhibited peritoneal tumor formation in L1210 lymphoma model [32]. Albini et al showed that daily administration of oral NAC in nude mice subcutaneously inoculated with immortalized Kaposi's sarcoma cells, resulted in a dramatic inhibition of tumor growth [33]. Simard et al found that cotreatment with intraperitoneal NAC synergistically enhanced antitumoral activity of shark cartilage extracts (SCE) in mice orthotopically implanted with mouse glioblastoma cells [34]. Investigating the utility of antioxidants as adjuncts to chemotherapy in colorectal cancer, Bach et al found that intraperitoneal administration of NAC enhanced the efficacy of 5-FU against HCT-15 tumor xenografts [35]. In a similar attempt towards the management of oral cancer, Lee et al demonstrated that intraperitoneal treatment of human tongue squamous carcinoma xenografts with NAC significantly reduced mean tumor volume by 33% [36]. In a recent study by Qanungo et al, while single agent chemotherapy with the first-line agent gemcitabine failed to inhibit pancreatic cancer growth, adjunct treatment with intraperitoneal NAC reduced tumor growth [37]. Sayin et al, however, reported that supplementing the diet with NAC and vitamin E increased tumor progression in mouse models of B-RAF- and K-RAS-induced lung cancer [38].

In the present study, consistent with the inhibitory effects of BR/NAC treatment on cell proliferation and growth in our *in vitro* [7] and *in vivo* (the present study) models is the significant reduction in the Ki-67 expression indices of the peritoneal tumors of either model in response to treatment. In agreement, Seril et al found that NAC treatment of a murine model of colorectal cancer resulted in a reduction in proliferating cell nuclear antigen (PCNA) index [39]. Likewise, Albini et al observed that Ki-67 and PCNA markers were significantly lower in Kaposi's sarcomas from NAC-treated mice compared to the untreated control [33]. In another study by Balansky et al, NAC significantly decreased PCNA in mouse lung tumors [40]. More recently, Poncin et al demonstrated that intraperitoneal NAC treatment of animal models of propylthiouracil (PTU)- or perchlorate-induced goitrogenesis significantly decreased the PCNA indices in both types of goiter [41].

Apart from revealing the inhibitory effects of BR/NAC on tumor growth, this animal study also indicated, consistent with our *in vitro* observations [7], that BR/NAC therapy remarkably diminished the tumor production of the membrane-associated mucin MUC1, as well as of the secreted mucins MUC2 and MUC5AC.

In keeping with the present data, mucoregulatory activity of NAC has been demonstrated in different experimental models. Rogers et al consistently observed that oral NAC inhibited cigarette smoke-induced mucin hypersecretion [22] in the rat airways. With respect to the secreted, gel forming mucins, NAC treatment was shown to hamper the expression of MUC5AC in lung carcinoma cells [18, 23]. In another study on the bleomycin-induced pulmonary fibrosis model, Mata et al found that orally administered NAC diminished MUC5AC expression in the rat airway epithelium [20]. Consistently, increased expression of MUC5AC induced by allergen challenge in rats was abolished by NAC [19]. Dharajiya et al found that coadministration of NAC and ascorbic acid reduced pollen challenge–induced mucin production in mice by 10 times [42]. Likewise, intraperitoneal NAC pretreatment of ovalbumin-sensitized and challenged mice significantly decreased bronchial mucin production [21]. Mata et al later indicated that MUC5AC expression induced by viral infections of the human respiratory epithelial cells was inhibited by NAC *in vitro* [43]. Similarly, Urashima et al found that topical administration of NAC significantly lowered the amounts of conjunctival and corneal mucins in the rabbit eyes [24].

In accord with tumor mucin-depleting activity of the treatment documented in the present study, BR/NAC was found in a parallel study in our Department to effectively disintegrate PMP-secreted mucin gels [13]. In agreement, a number of case reports provide evidence that supports the benefits of NAC-induced mucolysis in hepatobiliary conditions [16, 17]. Taken together, mucoregulatory and mucolytic properties of BR/NAC combination therapy represent a hallmark of this treatment that could be utilized to improve the efficacy and practicability of the conventional therapies for mucin-expressing tumors. Hence, utility of BR/NAC as a dual action adjunct in novel locoregional modalities could not only enhance microscopic cytoreduction attempted by chemotherapy, but also holds promise for *in situ* mucolysis in the context of mucin-secreting tumors.

Lastly, safety assessment of this experimental treatment revealed no evidence of adverse effects. Despite the fact that a standard toxicology study is yet to be conducted, the present study mimics a dose escalation study and provides a preliminary insight into the safety of the treatment.

Literature review consistently confirmed that BR and NAC are known to be generally safe agents. Our department previously reported that intraperitoneal administration to nude rats of BR/NAC, 4 times over 48 hours yielded no adverse effects [13]. With respect to the safety of the intraperitoneal treatment of animal models with BR, Baez et al reported no visible side effects after administration of BR at doses ranging from 1 to 50 mg/kg [44]. Similarly, clinical and gross pathological evaluation by Romano et al in a murine model of colon cancer revealed no toxic effects [45]. As with BR and in line with our experience, NAC has been used safely for intraperitoneal treatment of different murine models of cancer by other investigators. In their experiment with murine melanoma model of lung metastasis, De Flora et al treated their nude mice with intraperitoneal injections of NAC at a dose of 1 g/kg/day [30]. In the study by Lee et al on human tongue squamous carcinoma, animals were treated with intraperitoneal NAC at a dose of 100 mg/kg/day for 20 days [36]. Likewise, in the recent study by Qanungo et al, a similar dose of NAC was used for 35 days as an adjunct to gemcitabine therapy of pancreatic cancer xenografts [37].

Taken together, the preclinical evaluation of the BR/NAC efficacy in two models of peritoneal dissemination of gastrointestinal cancers revealed the relevance and translatability of our *in vitro* findings in *in vivo* settings. In other words, this experimental treatment showed preclinical promise for locoregional treatment of these malignancies, representing a modality with dual effects on cancer cells and their mucin synthesis. In addition, the present study provided preliminary safety evidence in favor of the peritoneal use of BR/NAC, which needs to be confirmed in a separate toxicology study. Our results lay the basis for further evaluation of this treatment as an adjunct in combination with chemotherapy.

## MATERIALS AND METHODS

### Cell culture

MKN45, KATOIII and LS174T were purchased from American Type Culture Collection (ATCC, Manassas, VA, USA). These cell lines were authenticated by DNA short tandem repeat profiling, and experiments were carried out within 6 months of resuscitation. Cell lines were routinely assessed by cell morphology and their average doubling time. All cell lines were maintained in a humidified 5% CO2 incubator at 37°C in their respective medium as follows: MKN45 in RPMI-1640 medium, KATOIII in IMDM, and LS174T in EMEM (Invitrogen, Carlsbad, CA, USA). The culture media used were all supplemented with 10% fetal bovine serum (FBS) and 1% penicillin-streptomycin, with the exception of IMDM being supplemented with 20% FBS. The culture medium for LS174T was further supplemented with 2 mM Glutamine and 1% Non-Essential Amino Acids (All from Invitrogen, Carlsbad, CA, USA).

### Drug preparation

Bromelain and N-acetylcysteine were purchased from Sigma-Aldrich (St. Louis, MO, USA). For treatment, the stock solutions were freshly made, with BR and NAC being dissolved in relevant culture media, filtered and pH adjusted (applicable for NAC). For *in vitro* and *in vivo* studies, drugs were diluted with the cell line-specific medium and 0.9% sterile Saline, respectively, according to the final treating concentrations required. The treatment regimens used have been shown in Table 1.

**Table 1:** Bromelain and NAC concentrations used in single agent and combination treatment of gastrointestinal carcinoma

Cell line			
*In vitro*	Single BR (μg/ml)	Single NAC (mM)	Combination (BR + NAC)
**LS174T**	20, 50	5, 10	20 + 5, 20 + 10, 50 + 5, 50 + 10
**MKN45**	100, 200	5, 10	100 + 5, 100 + 10, 200 + 5, 200 + 10
**KATOIII**	50, 100	50, 100	50 + 50, 50 + 100, 100 + 50, 100 + 100
***In Vivo***	**Single BR (mg/kg)**	**Single NAC (mg/kg)**	**Combination (BR+NAC)**
**LS174T**	3, 6	300, 500	3 + 300, 3 + 500, 6 + 300, 6 + 500
**MKN45**	3, 6	300, 500	3 + 300, 3 + 500, 6 + 300, 6 + 500

BR, bromelain; NAC, N-acetylcysteine

### Periodic Acid-Schiff's (PAS) staining

MKN45, KATOIII and LS174T cells were seeded onto sterile glass coverslips for 72 hours. Cells were then treated for 48 hours. Upon completion of the treatment, the cells were fixed and then stained with periodic acid solution. Subsequently, cells were stained with Schiff's reagent which was followed by counter-staining with hematoxylin solution (Sigma-Aldrich, St. Louis, MO, USA). Cells were then mounted and subjected to microscopic examination using Leica DMLB microscope, DC200 digital imaging system (Leica Microsystems, Wetzlar, Germany) in at least eight different fields across the slides to analyze PAS positive mucosubstances stained rose to magenta with blue nuclei. ImageJ software (Research Services Branch (RSB), NIH, USA) was used for quantification of PAS-stained areas. For Staining of tissue sections, paraffin-embedded tissue sections were deparaffinized and then subjected to the same staining procedure.

### Immunocytochemistry

Cells were seeded onto sterile glass coverslips and maintained at 37°C. 72 hours post incubation, cells were treated with various concentrations of BR, NAC and the combinations for 48 hours. Upon completion of the treatment, the cells were fixed and coverslips were immersed in 1% bovine serum albumin for one hour. Cells were then incubated at 4°C overnight with monoclonal anti-MUC1, anti-MUC2 or anti-MUC5AC antibodies (Abcam, Cambridge, MA, USA). For negative control samples, no primary antibody was applied. After being rinsed, samples were incubated with the secondary antibody, Alexa Flour 488 goat anti-mouse IgG (Abcam, Cambridge, MA, USA), for 1 hour in dark. Cells were then counter-stained with propidium iodide and mounted. Slides were visualized by Olympus IX71 laser scanning confocal microscope (Olympus, Center Valley, PA, USA) and X60 oil immersion lens. The FluoView software (Olympus, Center Valley, PA, USA) was used to overlay the images.

### Western blotting

The effect of BR/NAC on mucin content of the cells was explored using Western blot analysis of the mucin expressions 48 hours post-treatment. Briefly, at the endpoints, cultured cells were homogenized and the protein concentrations were quantified by BioRad protein assay (Bio-Rad Hercules, CA, USA). After protein separation by SDS-PAGE and transfer to polyvinylidene fluoride membranes (Millipore, Billerica, MA, USA), the following primary antibodies were applied to the membranes according to the manufacturers' protocols: mouse monoclonal anti-MUC1, anti-MUC2 and anti-MUC5AC (Abcam, Cambridge, MA, USA). The membranes were then treated with appropriate horseradish peroxidase-conjugated secondary antibodies (Cell Signaling Technology, Danvers, MA, USA). A similar process was carried out for the GAPDH protein, as a loading control, using anti-GAPDH mouse monoclonal antibody (Sigma-Aldrich, St. Louis, MO, USA). The antigen-antibody reaction was digitized with ImageQuant LAS 4000 Biomolecular imager and ImageQuant software (GE Healthcare, Little Chalfont, Buckinghamshire, UK).

### Enzyme-linked immunosorbent assays (ELISA)

Cells were plated in 6-well tissue culture plates for 72 hours and treated for 48 hours. The culture medium was collected and centrifuged, and the levels of MUC2 and MUC5AC in the medium were detected by the specific detection kits (Biotrend Chemicals, Destin, FL, USA) as follows. Standards and samples were added to the supplied 96-well plates and incubated for 2 hours. Plates were then emptied and subsequently filled with Biotin-antibody and incubated at 37°C for 1 hour. Then, plates were washed and incubated with HRP-avidin at 37°C. After subsequent incubation with TMB Substrate, Stop Solution was added to plates and the optical density (OD) was determined using the PowerWaveX microplate reader (Bio-Tek Instruments, Winooski, VT, USA) set to 450 nm and 570 nm.

### Development of animal models

The animal study was conducted in accordance with the ethical standards and according to the Declaration of Helsinki and national and international guidelines and was approved by UNSW Animal Care and Ethics Committee (ACEC) (approval number: 13/86B and 12/121B). One hundred and eight, six-week old female athymic Balb C nu/nu mice (Biological Resources, University of New South Wales, Sydney, Australia) were used to develop two different models by MKN45 or LS174T cells (*N* = 54/model). Post acclimatization, an optimized number of cells (2 × 10^6^ MKN45 cells and 1 × 10^6^ LS174T cells) was intraperitoneally injected to animals and they were allowed to develop the peritoneal disease. Before the commencement of the treatment, mice were randomly assigned to one of the nine study groups. Intraperitoneally administered on an alternate day basis, treatment started on days 14 and 7 post-inoculation and lasted 12 and 17 days for MKN45 and LS174T models, respectively. Control group received the vehicle (0.9% Saline) only. Regular monitoring of animals continued during the treatment period using a standardized method [46]. In this regard, apart from body weight and abdominal circumference, parameters of general wellbeing and indicators of pain and distress classified into four categories, including general appearance, natural behavior, provoked behavior and body condition, were checked and recorded. Upon completion of the treatment, animals were euthanized by intraperitoneal injection of Lethabarb (Virbac, NSW, Australia). Post euthanasia, gross appearance of the peritoneal disease was examined and photographed, and peritoneal tumors were excised, counted and weighed. For further laboratory studies, tissues specimens from peritoneal tumors, small intestine, colon and liver were collected. Fixed specimens were then embedded in paraffin and 5 μm-thick sections were prepared for staining.

### Immunohistochemistry and scoring

The MUC1, MUC2 and MUC5AC primary antibodies were purchased from Abcam (Cambridge, MA, USA). Ki67 primary antibody was purchased from DAKO (Glostrup, Denmark). The sections were first deparaffinized and microwaved for antigen retrieval. Thereafter, the samples were incubated with 3% hydrogen peroxide and DAKO blocking buffer, respectively, followed by overnight incubation at 4°C with primary antibodies. Binding of the primary antibody was detected by incubating the sections with secondary antibody using EnVision Detection Systems Peroxidase/DAB reagents (DAKO, Glostrup, Denmark) for 30 min. The sections were then counterstained with hematoxylin and mounted. Tonsil tissue was used as negative control tissue for mucins and as positive control tissue for Ki67. Additional negative controls were also included wherein primary antibodies were replaced with antibody diluents. For scoring, using Leica DMLB Microsystems, immunohistochemical staining of samples was evaluated by at least two observers blinded to sample identity. Representative slides were photographed using Leica DC200 digital imaging system. With regard to mucin, samples were classified as positive if >5% of tumor cells were stained positive and otherwise as negative. Furthermore, semi-quantitative scoring was attempted using the average intensity of staining in combination with the percentage of the immunoreactive cells. For this purpose, intensity scores of 0 to 3, representing no, weak, moderate and strong intensity, respectively, were used along with a four-value quantity score defined as follows: score 0, 0–5% positive cells; score 1, 6–25% positive cells; score 2, 26–50% positive cells; score 3, 51–75% positive cells; score 4, 76–100% positive cells 76–100% positive cells [47]. For each sample, the average intensity and quantity scores for at least 5 high power fields were combined, yielding an 8-point immunohistochemical score ranging from 0 (no staining) to 7 (extensive, strong staining). For Ki-67, a positive nuclear stain was considered indicative of positive staining and the percentage of the positively stained cells among the total number of tumor cells in the area was accordingly calculated [48]. For H&E staining, sections were first stained in hematoxylin solution for 3 minutes, then immersed in working eosin solution (Sigma-Aldrich, St. Louis, MO, USA) for 2 minutes, and finally mounted and viewed under the microscope.

### Statistical analysis

All data presented are representative of three independent experiments performed in triplicate. Statistical analysis was conducted using GraphPad Prism 6 (GraphPad Software, Inc., La Jolla, CA, USA). Data are presented as mean ± SEM. Student's *t*-test was applied for unpaired samples. One-way analysis of variance (ANOVA) was used to determine the statistical differences between more than two groups and *p* values <0.05 were considered significant. Two-way analysis of variance (ANOVA) was used to examine the influence of two different independent variables on one dependent variable.
